# Accessing rural health services: Results from a qualitative narrative gerontological study

**DOI:** 10.1111/ajag.12694

**Published:** 2019-06-28

**Authors:** Stephen Neville, Sara Napier, Jeffery Adams, Kay Shannon

**Affiliations:** ^1^ Department of Nursing Auckland University of Technology Auckland New Zealand; ^2^ School of Clinical Sciences Auckland University of Technology Auckland New Zealand; ^3^ Shore & Whariki Research Centre Massey University Auckland New Zealand

**Keywords:** access, health services, older adults, primary health care, rural

## Abstract

**Objective:**

Explore how older adults’ talk about accessing rural community health services.

**Methods:**

A qualitative narrative gerontological approach explored issues related to accessing health services in their community. Semi‐structured digitally recorded individual interviews were undertaken with 32 community‐dwelling older people aged between 75 and 93 years. A narrative data analytic process was undertaken. The COnsolidated criteria for REporting Qualitative research guidelines were followed to ensure rigour in this study.

**Results:**

Three collective narratives resulted from the data analytic process: (a) “accessing local health services”; (b) “accessing specialist services”; and (c) “accessing emergency services.”

**Conclusions:**

Narrators identified a number of issues related to accessing rural health services. These included long waiting times, lack of continuity in care provision by doctors and difficulties accessing specialist and emergency services. Nurses were frequently cited as a reliable point of contact for these older people. Expansion of nursing roles would enhance the provision of rural health‐care services.

**Policy Impact**

There is no doubt that as people age, there is going to be an increase in the occurrence of health issues. In many countries, “ageing in place” is a focus of many governments. If this is to be fully realised, then the provision of health services must be available and appropriate for older people.

**Practice Impact**

In an environment where there are difficulties attracting and retaining medical staff, utilising the skills and expertise of nurses to provide a range of health services is appropriate. Supporting the development of advanced nursing practice roles, such as nurse practitioner, would ensure the timely and appropriate delivery of rural health services to older adults.

## 1 INTRODUCTION

Global ageing is a reality. Based on the World Health Organization predictions, many countries are preparing for unprecedented increases in the number of people aged 65 years and over.^1^ These increases are expected in both urban and rural environments. The impact of an ageing population is already being realised in rural environments with many communities having higher numbers of older people when compared with urban areas. This phenomenon can be explained by demographic trends such as inward migration of older people choosing to live rurally due to factors such as affordability, and outward migration of younger people seeking enhanced education and employment opportunities.^2^

Many governments have recognised the importance of older people continuing to live in their local communities, commonly known as “ageing in place” and the positive impact this has on health and well‐being.^3^ Governments see “ageing in place” as a way of managing escalating health‐care costs. Research identifies an increase in health issues including rates of non‐communicable diseases and disability in those aged 65 years and over.^4^ An increase in health issues increases demand for health services by older people.

Research indicates living in an environment providing access to reliable and appropriate health services is important to older adult’s feelings of security and sense of belonging.^5^ Previous studies have demonstrated that older people living in rural areas have poorer health outcomes, are more likely to experience poverty and are less likely to access health services when compared to older adults living in urban areas.^6^ In addition, findings from *The Rural healthy People 2020* American study identified that having access to necessary health services was the most important rural health priority.^7^

Neoliberal government‐driven ideologies have seen a reduction in acute and hospital services in rural environments.^8^ Consequently, primary health‐care services are generally first point of contact for older people when they are unwell. Studies have shown that geographic location and distance is a significant barrier to accessing health services.^9^ Travelling distances to access these services is costly and time‐consuming due to limited public transport options, particularly for those who do not drive.^10^

The utilisation of technology, such as telehealth‐based services, is becoming increasingly popular and is touted as the panacea for removing barriers to accessing health services for older people living in rural areas.^11^ The success to providing telehealth services includes being able to recruit and retain the required skill mix of health professionals needed to support the use of these technologies. Extant literature has identified that rural communities report high turnover rates in all health professional groups even though some communities offer financial and other employment‐related incentives.^12^

The provision of health services to rural communities presents many challenges, and these have the potential to negatively impact the health of older people. To date, little research has sought the views of rural‐dwelling older people about the issues they have in accessing health services. Gerontologists have identified that policy and services are frequently formulated without consulting with older consumers, including those living in rural areas.^13^

### 1.1 Objective

The objective of this study was to explore how older adults talk about accessing rural community health services.

## 2 METHODS

When writing up the findings of this project, the COnsolidated criteria for REporting Qualitative research (COREQ) was followed and incorporated into the study.^14^

### 2.1 Design

A qualitative design underpinned by narrative gerontology formed the methodological foundations of this study. Narrative gerontology allows for the exploration of how older adults experience their surroundings^15^ and is grounded in the philosophical assumptions associated with interpretivism.^16^ There are a multitude of definitions available to describe what narrative gerontology is and how its concepts can be deployed. However, most gerontological researchers agree that this approach, which has its origins in narrative inquiry, supports the telling of an older person’s day‐to‐day experiences in their own words.^17^ Privileging and foregrounding the voices of older adults have the potential to influence and change practice. Consequently, the utilisation of narrative gerontology enabled the exploration of how older adults talk about accessing health services in a rural community with the aim to inform future planning of health‐care services.

### 2.2 Setting

The study was undertaken in a small New Zealand rural town with a population of 3,909 at the time of 2013 census. Approximately 26% of the population is aged 65 years and over.^18^ The town acts as a service centre for local farming and residential communities. It offers a range of amenities including supermarkets, cafes, banks, retail, library, information centre and a range of health services.

### 2.3 Participants

A purposive sampling strategy was employed to access older adults who could contribute towards answering the research aim.^19^ The study was promoted through advertisements in community newspapers and poster displays on noticeboards where older adults were likely to see them, for example in libraries. Those who were interested in participating made telephone contact with a member of the research team. Inclusion criteria for this study were those people aged 75 years and older, who were able to participate in an interview of approximately one‐hour duration, lived independently in the area and accessed health services. Data were collected in 2015.

### 2.4 Data collection

Prior to being interviewed, participants were provided the opportunity to read the information sheet and have any questions answered before signing the consent form. A convenient time and place to meet and undertake the interview was negotiated. Digitally recorded semi‐structured in‐depth interviews were then undertaken with 32 people who met the inclusion criteria. All participants preferred to complete approximately a one‐hour interview in their own home. Examples of questions asked during the interview included “Tell me about the type of health services you access” and “Do you feel the health services provided in your community meet your needs. Why/why not”? Recruitment and interviewing continued until data saturation was reached. Interview data were transcribed verbatim by a transcriber who signed a confidentiality agreement. To ensure confidentiality, participants were given pseudonyms.

### 2.5 Ethical considerations

Ethical approval for this study was obtained first from the Massey University Human Ethics Committee (MUHECN 15/010) and then from the Auckland University of Technology Ethics Committee (AUTEC 15/100).

### 2.6 Data analysis

It is recognised that there are a multitude of ways to approach narrative analysis and therefore no set formula.^16^ The narrative analysis process undertaken was based on Brown and Addington‐Hall’s framework.^20^ Firstly, each of the transcripts was read multiple times by two members of the research team to develop an overall understanding of the text. Secondly, commonly occurring narrative segments evident across data sets relating to the research aim were identified. Through an iterative process, the narrative segments were reworked, conceptualised and presented as collective narratives. Collective narratives are recognised as a legitimate form of presenting narrative data.^21^ All aspects of the analytic process were iterative, inductive, data‐driven and focused on semantic content.^15^ A summary of the collective narratives was offered to participants.

## 3 RESULTS

A total of 32 older people (20 women and 12 men) agreed and participated in the interviews. Ages ranged from 75 through to 93 years. Fourteen participants were widowed, 15 were married, one person identified as single and two people were divorced. Participants had lived in the area on average 26 years. Following the data analytic process described above, three collective stories were identified each of which related to accessing particular types of health services. The collective stories identified were named as “Accessing local health services,” “Accessing specialist services” and “Accessing emergency services.” Verbatim quotations from the transcribed data are used to support the three identified collective stories and these are presented below.

### 3.1 Accessing local health services—local services do not always meet local need

Narrators in this study were proud of living in this rural community and as expected always responded positively about the health services that were available to them. However, this was always followed up with “but” or “however,” indicating there were issues with the local health services that did not meet their needs. A dominant pattern spoken in the narratives was the length of time it took to get an appointment to see either the doctor or the nurse.

Overall, I think we have a very good health system here. I am just two minutes from my doctor and my dentist, people that I’ve been with for 20 years, the familiarity of it is great and I like the convenience. They are just down the road. But it’s just that our doctors have quite long waiting lists. If you want to get a normal check‐up I think it’s usually 2 to 3 weeks and that is a bit annoying. (Patricia, 81 years)

This rural community also serves as a service centre for a substantial number of holidaymakers. This means in summer there is increased pressure on existing local health services due to an influx of tourists, resulting in longer waiting times to see a doctor. For Colin, long wait times became a problem when he hurt his back and could not get an appointment until the following Friday.

The services the local medical centre provides is top notch [very good]. I go there to see my doctor or the nurse and I use their podiatrist because I’ve got problems with my feet. However, this summer I had a sort of bad back and rung up to see what they could do for me and they couldn't see me until next Friday! What was I meant to do? To be fair the nurse rang me back and talked to me about it, she is a local and was a great help but the wait time was terrible. (Colin, 79 years)

There are challenges in rural communities recruiting and retaining all health professional groups including medical staff as can be seen in the following narrative. Nurses therefore play an important role not only in the provision of health and nursing services but also maintaining relationships with consumers of health services. Ted articulates this in the excerpt below:

District nurses came out when I had my cancer, they came out to see if I was alright and look at the catheter or whatever it was I had. I thought that was a very good service and I know them and really appreciated them keeping an eye on me. However, when you go for a general checkup or something it seems like you never see the same doctor twice if you need to go back. I don’t like that, I don’t feel like they know me. (Ted, 90 years)

### 3.2 Accessing specialist services—specialist services are too far away

Government policy in New Zealand identifies the provision of health care close to where a person lives is important and is a facilitator for older people to age well.^22^ While some specialist services were available in this rural community, there were many instances where older people had to travel to the city, as identified in the following collective narratives.

We do have some specialists who come up from [name of the city]. They come up from the city and you can make appointments to see them through the doctor without having to travel. But they don’t cover everything so there are still lots of health things we have to travel for … it’s such a long way to go. (Olive, 76 years)

For some, accessing specialist services was not a current issue due to their present good health; however, as they were long‐standing members of this rural community, they were looking to the future, to a time when they were more likely to need to access specialist services.

While I don’t have any health problems at the moment, my biggest worry is for when I’m older and need to go to hospital. It is such a long way away. If I suddenly got sick the doctors here would not be able to manage it. How would I get there and would I get there in time? I don’t know anyone in [name of the city] and don’t have any family close by … what would I do. (Maureen, 75 years)

For others, there is always hope that more health services will be provided locally. Ruth has trouble with her vision which requires specialist intervention; however, these services are some distance away and travelling this distance and being away from home is not her preference.

[Name of the hospital] is the nearest but they don’t do eyes so I have to go to [name of the hospital] which is even further away. The local nurses are fantastic you know … she said that with the way our population is growing it won’t be long before we have our own eye specialist here. That will be so good for me, I won’t have to travel so far. I don’t like going too far from home these days. (Ruth, 87 years)

### 3.3 Accessing emergency services—worrying about accessing emergency services

Due to geographical isolation, those living in rural areas are at increased risk of mortality especially as a result of trauma.^23^ This is due to a lack of 24‐hour medical services being available and the time delays in ambulances arriving. The following narrative identifies some of the challenges older people face should they need to access emergency services.

Once the doctor’s surgery closes we have nothing … there's nobody on duty so you have to call the ambulance. The ambulance takes time to reach you … otherwise you have to get yourself to the city. Well that would be fairly difficult when you're sick, so those things are a worry really. I feel anxious about what I would do and how I would manage should anything happen. (Colleen, 79 years)

Several participants had purchased medical alarm services so that should anything untoward happen an alarm would be activated in an emergency call centre and help dispatched. This paid service provided piece of mind, not only for older people, but also for their families/significant others.

… after nearly drowning, splitting my head and all the other complicated things that I’ve done to myself I really thought I’d better get a medic alert. I live alone you see and my family don’t live close by. I was starting to worry and so were my children that if anything happened, you know if I had an accident, that no one would know. I thought it was for the best. (Robert, 92 years)

While extolling the benefits of medical alarm services, participants were clear that if emergency services were needed, living in a rural environment meant they were still a significant distance from an acute hospital. Older people were realistic that even if emergency services did arrive, they still may die in transit to the hospital.

My alarm is great … if I do have a fall or something they phone and if I don’t answer they send someone around. I’ve got a key hidden in a box which they know where it is so they can get in. However, it still would take them a long time to get to me and then the hospital is a long, long way away. If anything happened I’d be too far away and the ambulance would take too long to get to me … I’d probably die. (Barbara, 81 years)

## 4 DISCUSSION

The aim of the present study was to explore how older adults talk about accessing health services in a rural community. Older adults in this study identified that access to all health services in their rural community is limited. This is despite primary health‐care services being regarded as fundamental to all communities. Many countries identify that a functioning and accessible primary health‐care system is integral to maintaining and improving the health and well‐being of all citizens including those who are older.^22^

Participants talked about how they were frequently faced with long wait times before being able to get an appointment to see a doctor. Wait times were influenced by a lack of medical professionals in relation to the number of people living in this community, combined with an influx of holidaymakers burdening already challenged health services during the summer holiday period. The collective narratives also reinforced the importance of older adults placed on being known by their health practitioner. This group of people did not appreciate seeing a different doctor each time they required medical services. Studies undertaken in rural Canada found that the provision of appropriate and consistent primary health‐care services to older communities was influenced by the ability to recruit and retain health professionals including physicians.^24^ Difficulties with recruitment and retention lead to the utilisation and reliance on “locum” or casual health professionals, a common feature in the provision of primary health care in rural areas.^25^

As evident in the narratives, nurses were reported as being central figures in the provision of health services, including being first point of contact when a doctor was not available, as well as providing a diverse range of health services both inside clinics and in community settings (eg house calls by district nurses). Nurses working in rural environments are frequently long‐standing members of their rural community and as such are able to provide the continuity of care that these participants desired.^26^ Consequently, the utilisation of advanced nursing practice roles, such as nurse practitioners, and other advanced nursing practice roles is integral to the provision of all rural health services that meet the needs of older people.^27^

Limited access to specialist and emergency services was also a noted collective narrative evident in the data. While some specialist services were available in this community, these were limited and required older people to travel significant distances for consultations for many specialist services. Travelling to access medical services is known to be hindered by limited public transport options, due to the person no longer driving, or lacking the confidence and skill to navigate a road journey into an urban centre. In New Zealand, after the age of 75 years drivers must undergo a medical assessment and, in some cases, undertake an on‐road driver safety test in order to be able to continue to drive.^28^ Consequently, older people who are no longer driving frequently rely on others or public transport, and this reliance can be problematic limiting accessibility to specialist and emergency services. For example, in a qualitative study, Walsh et al^29^ found that limited timetable schedules, distances to walk to bus stops and difficulty getting on and off buses were access barriers for older people living in rural communities. Unsurprisingly, research^30^ has also found travel times for older adults accessing medical specialists were significantly longer for those using public transport when compared to those travelling by car.

In order to mitigate the lack of emergency services, several participants narrated the purchase of medical alarm services. While this provided a level of consolation, all those interviewed were realistic of the limitations and issues associated with using a medical alarm should an emergency arise. For example, participants were worried they might die due to the length of time it would take emergencies services to reach them after the medical alarm was activated. Unsurprisingly, a retrospective cohort study of adult/older adult trauma deaths found that those who lived in rural areas had higher rates of preventable prehospital deaths than those living in urban areas.^31^

The increasing use of technologies including telemedicine and other information technologies has been touted as solutions to the challenges inherent in the provision of health care in rural communities. While the cost, in terms of time and money, for older adults living rurally to travel to access health services is reduced with the advent of telemedical services, several challenges remain. A systematic review of telehealth services in rural and remote Australia found that appropriate funding mechanisms, supported infrastructure, as well as the availability and expertise of health professionals were constitutive to the provision of rural health services.^9^ In addition, the provision of telehealth services can only occur if communities have access to the Internet that is both stable and reliable.

The study is limited by homogeneity of the participants, all of whom were of European descent. Consequently, the narratives of older Māori (indigenous people of New Zealand) and those from other ethnic groups were not captured. The research could have been further strengthened by incorporating an observational component as a data collection technique. This would enable researchers in real time to first hand observe the realities that older adults face when accessing rural health services.

## 5 CONCLUSIONS

Overall, the collective narratives from this study identify several challenges to older people trying to access rural health services. Therefore, these challenges impact on the health of this group of narrators. In an environment where there are limited resources and issues with attracting and retaining medical staff, it is appropriate to better utilise the skills and expertise of the nursing workforce. If governments are serious about supporting “ageing in place,” then they must provide health services that are both appropriate and accessible to older adults.

## CONFLICT OF INTEREST

The authors declare no conflicts of interest.
